# Molecular Detection of Secreted Aspartyl Proteinases (Saps) From Dental Isolates of Candida albicans and Targeting With Psidium guajava Biocompounds: An In Vitro and In Silico Analysis

**DOI:** 10.7759/cureus.49143

**Published:** 2023-11-20

**Authors:** Nehal Safiya S, A. S. Smiline Girija, Vijayashree J Priyadharsini

**Affiliations:** 1 Department of Microbiology, Saveetha Dental College and Hospitals, Saveetha Institute of Medical and Technical Sciences (SIMATS) Saveetha University, Chennai, IND

**Keywords:** health, myricetin, in-silico, sap, psidium guajava, candida albicans

## Abstract

Introduction

*Candida albicans* (*C. albicans*) is an opportunistic yeast-like fungus and is considered a functional biome of the oral and gut microbiomes. The *sap* gene and its types play a vital role in the pathogenesis of *C. albicans*. The emergence of resistance traits is a major problem, and targeting the same with alternative medicines has sparked renewed interest in recent years.

Objectives

This study is thus aimed at detecting the frequency of *sap* gene types in the clinical isolates of *C. albicans* and evaluating the antifungal effect of the crude methanolic extract of *Psidium guajava *(*P. guajava*). Further in silico assessments will assess the inhibitory effect of six compounds of *P. guajava* against the Sap protein.

Materials and methods

*C. albicans* was characterized phenotypically in 20 patients with root caries, and the *sap* gene was detected by PCR. The crude methanolic extract was prepared, and its antifungal efficacy was evaluated by the agar well diffusion method. Auto-docking was performed to assess the best compound based on the docking and overall interactions.

Results

Six isolates were identified as *C. albicans* and *sap *gene types 1-3 were detected in the four strains. *P. guajava* methanolic extracts showed a promising antifungal effect at varying concentrations. In silico analysis showed myricetin possessing the maximum number of hydrogen bonds and high docking energy with one violation.

Conclusion

The study concludes that *P. guajava* has a promising inhibitory effect against *C. albicans* with myricetin as the best compound to target the *sap* gene of *C. albicans*. However, further experimental studies are to be considered for its effectiveness in treating the infections caused by *C. albicans*.

## Introduction

The opportunistic yeast-like fungus *Candida albicans *(*C. albicans*) is considered one of the important members of the functional biome of the oral and gut microbiome [1]. It contributes to virulence, specifically in immunocompromised patients, affecting the oral-mucosal tissues [2]. In dental patients, it is frequently detected in root caries and is also a potent biofilm formed in dental implants [3]. Many virulent proteins, such as secreted aspartyl proteinases (Saps), phospholipase B enzymes, and lipases, are associated with the disease establishment by *C. albicans*. Amidst these, the Saps are encoded by nearly 10 different genetic determinants and exhibit different disease forms.

Saps are considered the most important virulence factor in *C. albicans*, playing a potent role in adherence to the tooth surfaces [4]. The sequence homology of the Saps shows variations, and thus based on the amino-acid sequences, they are categorized into 10 types. They are known to express themselves in a differential manner based on the host and the habitat. The expression and presence of the *sap* types vary in different patients and in animal models [5,6]. The virulence of *sap* in the establishment of various systemic infections has also been documented [7].

Amidst the virulence of Saps, *C. albicans *also emerges as resistant traits and is thus a major challenge in treating complicated recalcitrant infections. Plant-based medicines and their bio-compounds have been implemented to tackle the same, and many bioactive compounds have been discovered as novel drugs. Among many plant-based compounds, *Psidium guajava *(*P. guajava*), the common guava, is an evergreen shrub or a small tree that is native to tropical countries [8]. In traditional medicine, *P. guajava* has been used to treat a wide variety of systemic and infectious diseases [9].

*P. guajava *(guava) is a well-known tropical tree with medicinal properties against different systemic illnesses [10]. The leaf of *P. guajava* has wide applications in oral ulcers and gingivitis [11]. Its fruit is rich in vitamins and various minerals with potent antioxidant, anti-inflammatory, and anti-cancer properties [12,13]. Structural elucidation of *P. guajava *extracts shows many vital phytocompounds with high medicinal values [14].

With this background, the present investigation is aimed at evaluating the detection of *sap* from *C. albicans* in patients with root caries, together with the inhibitory effect of the methanolic crude extracts of *P. guajava.* Further in silico evaluation is designed to assess the drug-ligand interactions of the *sap* with the six bioactive compounds from* P. guajava*.

## Materials and methods

Study setting and characterization of *C. albicans*

This is a prospective observational three-month period of study (April 2022 to June 2022) conducted in the Department of Microbiology at Saveetha Dental College and Hospitals. Institutional review board approval, ethical clearance, and informed consent were obtained prior to the start of the work (SRB/SDC/UG-2097/21/MICRO/060; IHEC/SDC/UG-2097/21/MICRO/601). Twenty patients with typical root caries, as examined by a dentist, were selected for the study. The carious scrapings were excavated and collected in sterile Sabouraud dextrose broth and were immediately transferred to the microbiology laboratory. The samples were inoculated onto sterile Sabouraud dextrose agar and also in HiCrome *Candida* differential agar and incubated at 37°C for 24 hours. After incubation, colonies were identified using the color of the colonies, colony morphology, and gram staining for the preliminary identification of *C. albicans*.

Genotypic characterization of *sap *gene in *C. albicans*

From the Sabouraud dextrose agar, fresh cultures of *C. albicans *were retrieved, and the genomic DNA was extracted using the manufacturer’s instructions (Qiagen kit, Hilden, Germany). *sap* types from one to seven were detected by performing PCR with 15 µl of the reaction mixture and specific primers of *sap* (0.1 µl of 100 pmol/ml concentration) (Table 1).

**Table 1:** Primers used to detect the sap genes from the clinical isolates of C. albicans

Gene target	Primers	Annealing temperature	Amplicon size
sap1	5′-TCAATCAATTTACTCTTCCATTTCTAACA-3′ 5′-TCAATCAATTTACTCTTCCATTTCTAACA-3′	58℃	161
sap2	5′-AACAACAACCCACTAGACATCACC-3′ 5′-TGACCATTAGTAACTGGGAATGCTTTAGGA-3′	58℃	178
sap3	5′-CCTTCTCTAAAATTATGGATTGGAAC-3′ 5′-TTGATTTCACCTTGGGGACCAGTAACATTT-3′	58℃	231
sap4	5′-CATTCATTCCTTTAATACCGACTATC-3′ 5′-GGTAACAAACCCTGTAGATCTTTTAAC-3′	58℃	156
sap5	5′-CATTCATTCCTTTAATACCGACTATC-3′ 5′-GGTAACAAACCCTGTAGATCTTTTAAC-3′	58℃	181
sap6	5′-CATTCATTCCTTTAATACCGACTATC-3′ 5′-GGTAACAAACCCTGTAGATCTTTTAAC-3′	58℃	206
sap7	5′-GAAATGCAAAGAGTATTAGAGTTATTAC-3′ 5′-GAATGATTTGGTTTACATCATCTTCAACTG-3′	58℃	196

Amplification for 35 cycles was performed at an annealing temperature of 58°C. The amplicon sizes were visualized on a 1.5% agarose gel with EtBr, and the size was assessed with a 1.5 Kbp DNA ladder.

Preparation of the *P. guajava* extract

*P. guajava *fruits were obtained from local market vendors, and using sterile distilled water, the fruits were washed three times properly to remove the dust. Using sterile blades, the fruits were cut into small pieces, shade-dried, and ground into a fine powder. Ten grams of *P. guajava* powder were then weighed and added to 100 ml of methanol, which was kept for one week at room temperature with intermittent mixing. After one week, the crude filtrate was collected by filtering the mixture on Whatman No. 1 filter paper, followed by evaporation and complete drying. The crude yield was stored at 4°C until further bioassay procedures.

Antifungal bioassay

A varying concentration of *P. guajava* (100 mg, 50 mg, 25 mg, 12.5 mg, and 6.25 mg) was prepared with 1 ml of dimethylsulfoxide and vortexed. Lawn cultures of *C. albicans* were made onto the sterile Saboraud dextrose agar, and wells were punctured with a sterile agar cutter. About 50 μl of the diluted extract was added to the appropriate wells, followed by a 48-hour incubation period at 37°C. The bioassay was performed in triplicates, and the mean value of the zone of clearance was recorded.

Sap protein retrieval and optimization

The crystal structure of *sap1* was retrieved from the UniProt data bank with added hydrogen atoms to optimize it. Electronic charges were assigned to the Sap protein using the AutoDock tool version 1.5.6 (Center for Computational Structural Biology, California, USA), and the 3D structure of *sap* was visualized by the RASMOL tool (http://www.openrasmol.org/).

*P. guajava* ligand selection after optimization

Five bio-compounds from *P. guajava*, viz., avicularin, apigenin, hyperin, myricetin, chlorogenic acid, and fluconazole as controls, were selected using the ChemSketch software (Advanced Chemistry Development, Ontario, Canada). Using the open babel molecular converter, suitable conformations were done for the ligands to be optimized and were finally stored in PDB and saved in a .mol file.

Molinspiration molecular parameter evaluation of the ligands

The suitability of the selected ligands for their drug properties, bioavailabilities, membrane permeability, molecular weight, and further evaluation of the violations and ADME properties were assessed using a molinspiration tool and based on Lipinski's rule of five. The compounds were assessed for hydrogen donors and acceptors, lipophilic properties such as miLogP and TPSA values, and for the N-atoms. The selected compounds were also evaluated for drug likeliness scores for their ability to bind with the human G protein-coupled receptors, for their ability to modulate the ion channels, and finally their inhibitory properties for kinases, nuclear receptors, proteases, and other enzymes.

Drug ligand interactions of *P. guajava* compounds with *sap1*

The chemical affinities via various interactions between the selected compounds and *sap1* were evaluated using the AutoDock tool. The chemical interactions formed were visualized using the Discovery studio visualizer (Dassault Systèmes, Vélizy-Villacoublay, France), with further observation of their relative stabilities using the binding energies and the docking scores.

## Results

Identification of *C. albicans* from the clinical samples

**Figure 1:**
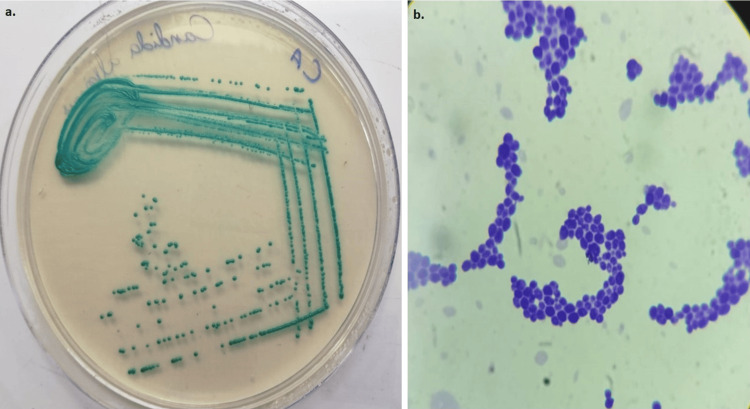
Phenotypic characterization of C. albicans from the clinical samples. (a) C. albicans growth on HiCrome Candida differential agar. (b) Gram staining showing gram-positive budding cells of C. albicans

Eleven isolates of *C. albicans* were identified from the specimens based on the typical creamy white moist colonies on the Sabouraud dextrose agar plate and greenish blue-colored colonies on the HiCrome *Candida* differential agar and gram staining that showed the gram-positive oval cells (Figure 1).

**Figure 2:**
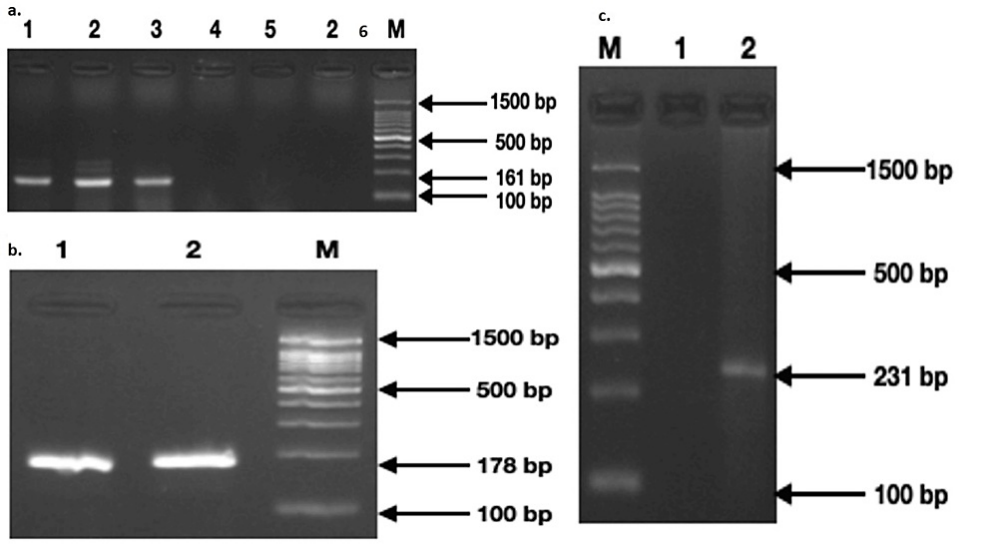
PCR amplification of the sap types 1, 2, and 3 from the clinical strains of C. albicans. (a) Electropherogram of sap1 with an amplicon size of 161bp in lanes 1, 2, and 3. (b) sap2 with an amplicon size of 178bp in lanes 1 and 2. (c) sap3 gene product of size 231bp in lane 2 (1.5k bp marker lane (M))

Genotypic characterization of *sap* gene types showed three isolates (27%) with the presence of *sap1*, two isolates (18%) with *sap2*, and one isolate (0.09%) with *sap3* (Figure 2). None of the strains showed the presence of *sap *types 4, 5, 6, and 7.

Antifungal activity of *P. guajava* methanolic extract

**Figure 3:**
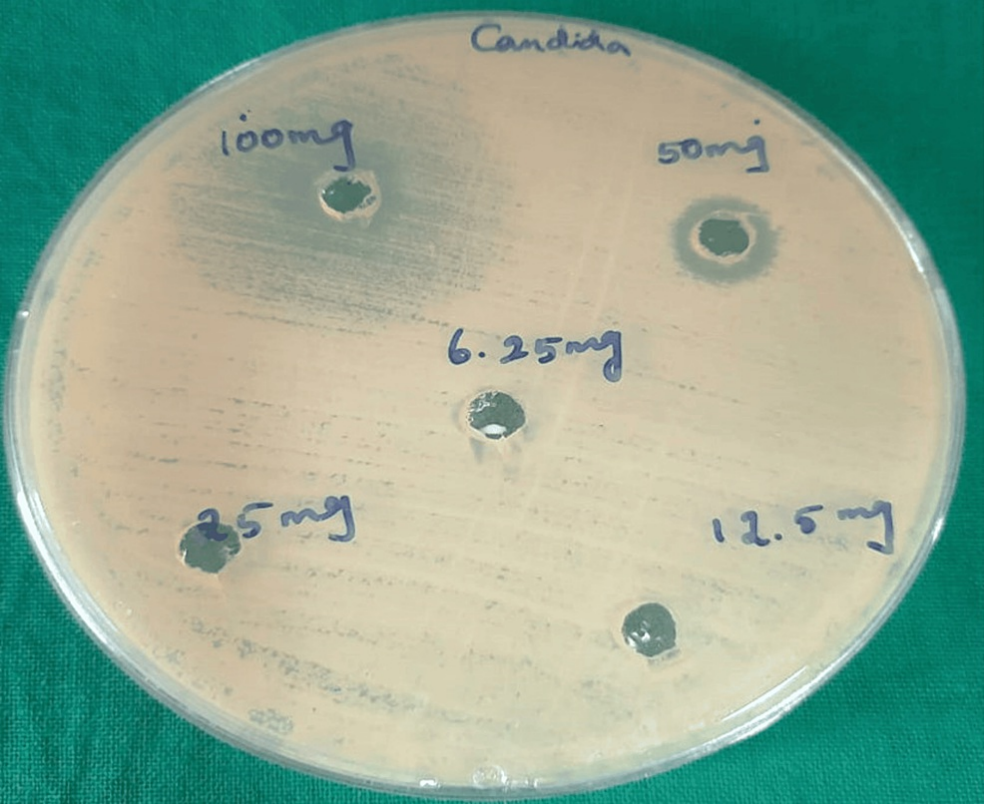
Antimicrobial effect of the crude methanolic extracts of P. guajava at varying concentrations (100, 50, 25, 12.5, and 6.25 mg) against the clinical strains of C. albicans

The methanolic extract of *P. guajava* yielded 34 mg of crude extract from 100 gm of the dried fruit powder. The agar well diffusion assay for the antifungal activity showed a promising antifungal effect against all the isolates tested, with a zone size of 19 mm for 100 mg and 12 mm for 50 mg/ml concentrations and no effect with concentrations of 25, 12.5, and 6.25 mg/ml concentrations (Figure 3).

*Sap1* structure and molinspiration results

**Figure 4:**
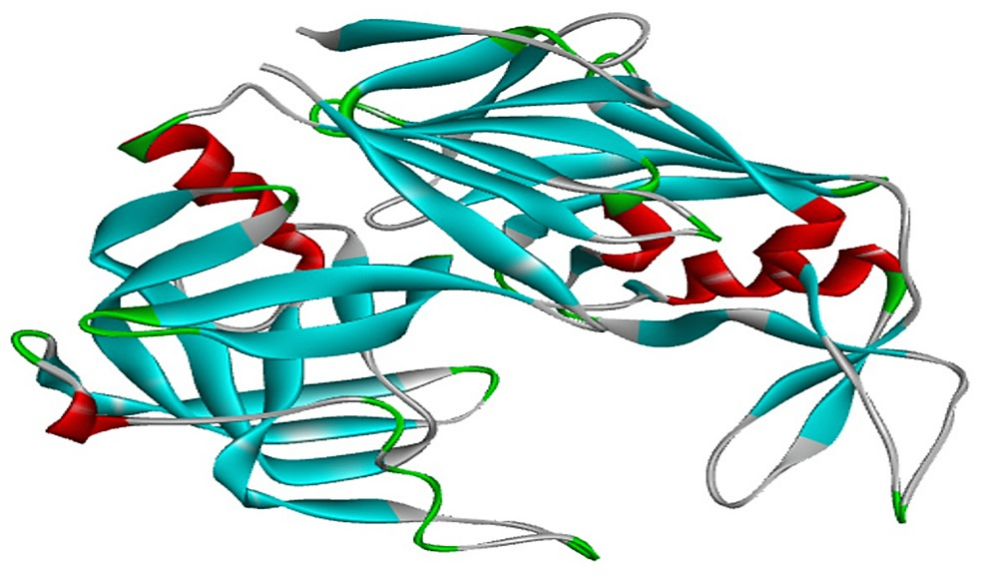
3D structure of sap1 visualized using RASMOL. Red indicates the Alpha-helix; blue indicates Beta sheets; white indicates the turns

*Sap1* from the *C. albicans* was successfully retrieved with the structure ID 2QZW-A-chain, and the 3D structure retrieved using RASMOL is given in Figure 4.

Molinspiration results showed zero violations of Lipinski's rule of five, and most of the selected compounds showed good interactive scores for the drug properties (Table 2).

**Table 2:** Molinspiration assessments for the selected P. guajava bio-compounds for the drug likeliness properties (set default score >0.2)

Compound name	Avicularin	Apigenin	Hyperin	Myricetin	Chlorogenic acid	Fluconazole
Hydrogen bond donor	7	3	8	6	6	1
Hydrogen bond acceptor	11	5	12	8	9	7
miLogP	0.80	2.46	-0.36	1.39	-0.45	-0.12
Rotatable bonds	4	1	4	1	5	5
nViolations	2	0	2	1	1	0
Topological polar surface area (Ǻ)	190.28	90.89	210.50	152.58	164.74	81.66
Volume	347.36	224.05	372.21	248.10	296.27	248.96
N atoms	31	20	33	23	25	22

The bioactivity scores assessed >0.03 were also promising for the selected ligands under study (Table 3).

**Table 3:** Bioactivity scores (set default >0.3) for the selected P. guajava bio-compounds under study

Compounds	Avicularin	Apigenin	Hyperin	Myricetin	Chlorogenic acid	Fluconazole
Human G protein-coupled receptor ligand	0.20	-0.07	0.06	-0.06	0.29	0.04
Ion channel modulator	-0.04	-0.09	-0.04	-0.18	0.14	0.01
Kinase inhibitor	0.21	0.18	0.13	0.28	-0.00	-0.09
Nuclear receptor ligand	0.06	0.34	0.20	0.32	0.74	-0.23
Protease inhibitor	-0.01	-0.25	-0.06	-0.20	0.27	-0.09
Enzyme inhibitor	0.53	0.26	0.42	0.30	0.62	0.03
Binding energy	-6.16	-6.84	-6.47	-6.9	-5.04	-5.04

In the molinspiration evaluations, apigenin and fluconazole showed zero violations, followed by myricetin and cholorogenic acid with one violation. Hyperin and avicularin showed two violations. The selected compounds showed a good TPSA value for all the compounds except apigenin. TPSA for fluconazole was also detected less. miLogP is an essential predictor for the lipophilic property, and it was toward the best score. The drug likeliness for the nuclear receptor and the enzyme inhibitor values are >0.3, and the selected compounds showed a drug likeliness score of above 0.2. The larger the drug likeliness values of the compounds, the more active they are, and they are thus considered for further docking analysis.

Results of the *sap1* interactions with the *P. guajava* bio-compounds

**Figure 5:**
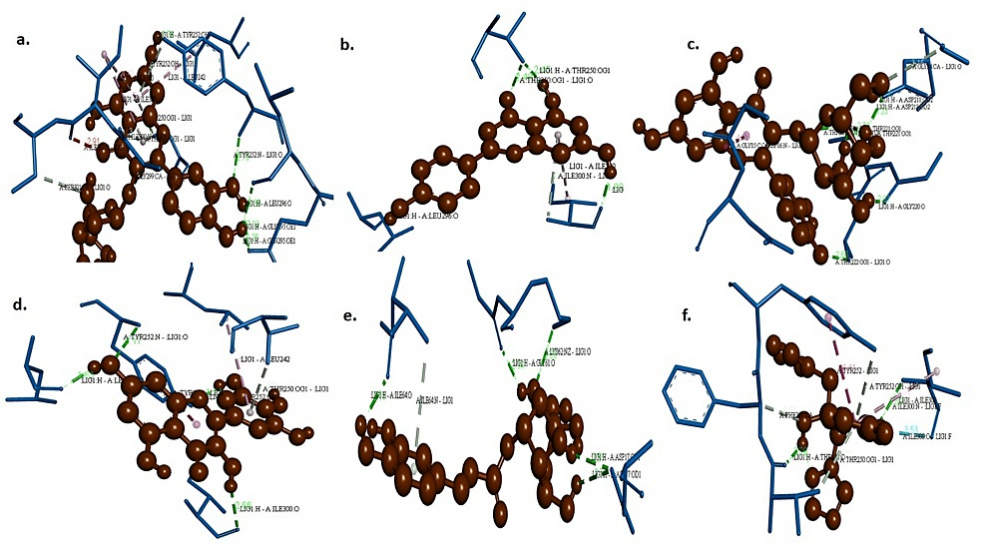
Visualizing hydrogen interactions between sap1 with (a) avicularin, (b) apigenin, (c) hyperin, (d) myricetin, (e) chlorogenic acid, and (f) fluconazole Source: images were created by the author

The binding energies and docking scores obtained between *sap1* and the compounds avicularin, apigenin, hyperin, myricetin, chlorogenic acid, and fluconazole were promising and are shown in Figure 5.

Myricetin showed four hydrogen bonds with a docking score of -6.9. It was followed by apigenin at -6.84, hyperin at -6.47, and avicularin at -6.16. The overall interactions for the scores of other interactions like van der Waals, alkyl-ᴫ, ᴫ-alkyl, p-sulphur, and p-alkyl were also highly promising for the compounds to be further considered as suitable drug candidates.

## Discussion

The opportunistic yeast *C. albicans* has been documented as the most frequent source of hospital-acquired infections [15]. *C. albicans* has been reported to cause two major types of infections, such as fatal systemic infections and superficial infections. It is documented that 75% of human beings possess *C. albicans* and other *Candida* species to a lesser level in the oral cavity, with refractory oral infections in immune-compromised individuals [16]. Resistance in *C. albicans* aids in the progression of the disease and affects the treatment regimens [17].

*C. albicans* is known for its production of vital virulent factors, and the *sap* gene family is differentially expressed in the flipped states of the candidal cells and is a suitable target for novel drug design [18]. *sap* was thus chosen for the present investigation, and we aimed to assess the frequency of the *sap* types in the clinical isolates of *C. albicans* from root caries. In the present study, we found the association of *C. albicans *in root caries and the prevalence of *sap* types 1-3 in the clinical isolates. The other *sap* types were not detected in the present study. In contrast, a study by Vita et al. documented the specific expression of *sap* types 4-6 from *C. albicans* from HIV-positive cases and from the normal flora of healthy individuals [19]. At the same time, *sap* types 1-3 have been shown to be expressed in different candidal species, and their frequency correlates with our study [20].

The emergence of resistant traits is a major challenge for all clinicians, and alternative medicine is being implemented to combat the menace of resistant *C. albicans* strains. In this context, in the present study, crude extracts of *P. guajava* were checked against the clinical isolates of *Candida* species. The antifungal efficacy was moderate in the present study, and many earlier studies have documented only a minimal effect, stating that *Psidium* compounds may have an inhibitory effect on the virulence of *Candida *sp. rather than antifungal effects [21].

In silico-based approaches to determining novel compounds from natural plants have sparked renewed interest in recent years. Many computational-based studies have been performed to assess the different bioactivities of various sources and were found to be promising [22-24]. Health benefits from *P. guajava* are due to a plethora of phytochemicals, such as quercetin, avicularin, apigenin, guaijaverin, kaempferol, hyperin, myricetin, gallic acid, catechin, epicatechin, chlorogenic acid, epigallocatechin gallate, and caffeic acid. In this note, six compounds were chosen for the docking analysis in the present study, and fluconazole was added as a control. Molinspiration parameters showed the drug properties, where apigenin and fluconazole showed no violations, followed by one violation by myricetin and chlorogenic acid. TPSA is considered a very useful analysis to evaluate the drug absorption and miLopgP for the oral bioavailability of the selected compounds. Based on this, hyperin showed the highest TPSA value (210.50), followed by avicularin (190.28) in comparison with the TPSA standard of 140 Å. As it is a useful descriptor to analyze the absorption of any selected drugs or for their bioavailability and transportation ability, the TPSA scores were very promising for the selected compounds. Due to the high values, it is also predictable that there may be smooth and efficient receptor binding as well.

Computational analysis of the interactions between the bioactive compounds and *sap1* showed promising docking interactions. Myricetin formed the highest number of hydrogen bonds with a binding energy of -6.9, followed by hyperin with three bonds and a docking energy of -6.47. Though apigenin did not violate the rules, it showed one hydrogen bond with a docking score of -6.84, higher than hyperin. The control fluconazole, also showing no violations, exhibited only one hydrogen bond with a docking score of -5.04. The compound myricetin may be thus taken for further purification, and it can be translated as a suitable drug of choice to treat the local and systemic infections caused by *C. albicans. *With all these assessments, myricetin was considered the best compound of choice, which may be further experimented with for its suitability as a drug candidate to treat candidal infections.

Limitations of the study

The present study has its own limitations, where the purification analysis was not performed and the antifungal effect of the purified compounds was not assessed. However, the study is in progress, and we have determined to evaluate the same in the near future, progressing further with the cytotoxicity and preclinical trials.

## Conclusions

We conclude this study with the association of *C. albicans* with root caries and the frequency of the sap gene types in the clinical strain of *C. albicans*. *P. guajava* and their bio-compounds possess promising antifungal activity against the sap-producing strains of *C. albicans*. Upon computational docking analysis, myricetin is the best compound characterized by the *P. guajava* compounds, which may require further experimental validation for its in vivo inhibitory and toxicity studies to develop myricetin as an alternative to the existing antifungal agents.

## References

[1] Girija AS, Ganesh PS (2022). Functional biomes beyond the bacteriome in the oral ecosystem. Jpn Dent Sci Rev.

[2] Hosain Pour A, Salari S, Ghasemi Nejad Almani P (2018). Oropharyngeal candidiasis in HIV/AIDS patients and non-HIV subjects in the Southeast of Iran. Curr Med Mycol.

[3] Yoo YJ, Kim AR, Perinpanayagam H, Han SH, Kum KY (2020). Candida albicans virulence factors and pathogenicity for endodontic infections. Microorganisms.

[4] Li W, Yu D, Gao S, Lin J, Chen Z, Zhao W (2014). Role of Candida albicans-secreted aspartyl proteinases (Saps) in severe early childhood caries. Int J Mol Sci.

[5] Naglik JR, Newport G, White TC (1999). In vivo analysis of secreted aspartyl proteinase expression in human oral candidiasis. Infect Immun.

[6] Schaller M, Schäfer W, Korting HC, Hube B (1998). Differential expression of secreted aspartyl proteinases in a model of human oral candidosis and in patient samples from the oral cavity. Mol Microbiol.

[7] Lian CH, Liu WD (2007). Differential expression of Candida albicans secreted aspartyl proteinase in human vulvovaginal candidiasis. Mycoses.

[8] Naseer A, Hussain S, Naeem N, Pervaiz M, Rahman M (2018). The phytochemistry and medicinal value of Psidium guajava (guava). Clin Phytoscience.

[9] Nwinyi O, Chinedu SN, Ajani OO (2008). Evaluation of antibacterial activity of Psidium guajava and Gongronema Latifolium. J Med Plants Res.

[10] Mukhtar HM, Ansari SH, Bhat ZA, Naved T, Singh P (2006). Antidiabetic activity of an ethanol extract obtained from the stem bark of Psidium guajava (Myrtaceae). Pharmazie.

[11] Rahim N, Gomes DJ, Watanabe H, Rahman SR, Chomvarin C, Endtz HP, Alam M (2010). Antibacterial activity of Psidium guajava leaf and bark against multidrug-resistant Vibrio cholerae: implication for cholera control. Jpn J Infect Dis.

[12] Smith RM, Siwatibau S (1975). Sesquiterpene hydrocarbons of Fijian guavas. Phytochem.

[13] Lok B, Babu D, Tabana Y, Dahham SS, Adam MA, Barakat K, Sandai D (2023). The anticancer potential of Psidium guajava (guava) extracts. Life (Basel).

[14] Liu H, Wei S, Shi L, Tan H (2023). Preparation, structural characterization, and bioactivities of polysaccharides from Psidium guajava: a review. Food Chem.

[15] Mayer FL, Wilson D, Hube B (2013). Candida albicans pathogenicity mechanisms. Virulence.

[16] Akpan A, Morgan R (2002). Oral candidiasis. Postgrad Med J.

[17] Ushanthika T, Smiline Girija AS, Paramasivam A, Priyadharsini JV (2021). An in silico approach towards identification of virulence factors in red complex pathogens targeted by reserpine. Nat Prod Res.

[18] Ranasinghe A, Girija AS, Priyadharsini JV (2020). Targeting the secreted aspartic proteinase (SAP-1) associated with virulence in C. albicans by C. cassia bio-compounds: a computational approach. J of Pharm Res Int.

[19] Meylani V, Sembiring L, Fudholi A, Wibawa T (2021). Differentiated sap (4-6) gene expression of Candida albicans isolates from HIV-positive patients with oral candidiasis and commensals in healthy individuals. Microb Pathog.

[20] Soroosh Dabiri, Masoomeh Shams-Ghahfarokhi, Mehdi Razzaghi-Abyaneh (2016). SAP(1-3) gene expression in high proteinase producer Candida species strains isolated from Iranian patients with different candidosis. J Pure Appl Microbiol.

[21] Morais-Braga MF, Carneiro JN, Machado AJ (2017). Phenolic composition and medicinal usage of Psidium guajava Linn.: antifungal activity or inhibition of virulence?. Saudi J Biol Sci.

[22] Kumar S, Manoharan S, Geetha Geetha (2021). Evaluation of efficacy of cinnamon oil as a root canal disinfectant - an in vitro study. Int J Dentistry Oral Sci.

[23] Sankar S (2022). In silico design of a multi-epitope Chimera from Aedes aegypti salivary proteins OBP 22 and OBP 10: a promising candidate vaccine. J Vector Borne Dis.

[24] Sunitha J, Krishna S, Ananthalakshmi R, Jeeva JS, Girija AS, Jeddy N (2017). Antimicrobial effect of leaves of Phyllanthus niruri and Solanum nigrum on caries causing bacteria: an in vitro study. J Clin Diagn Res.

